# Efficacy of diode laser on healing in frenectomy compared to conventional frenectomy with scalpel

**DOI:** 10.3205/dgkh000583

**Published:** 2025-09-23

**Authors:** Karthik Shunmugavelu, Evangeline Cynthia Dhinakaran

**Affiliations:** 1 Department of Dentistry, PSP Medical College Hospital and Research Institute Tambaram Kanchipuram, Tamil Nadu District, India; 2 Department of Pathology, Sree Balaji Medical College Hospital and Research Institute, Chrompet, Chennai, Tamil Nadu, India

**Keywords:** frenectomy, diode laser, scalpel, postoperative pain, wound healing, patient satisfaction

## Abstract

**Introduction:**

Frenectomy is a routine oral surgical procedure performed to excise an abnormal frenum attachment in order to enhance oral function and esthetics. Diode laser has become more popular than conventional scalpel methods, due to promises of decreased postoperative pain, less bleeding, and quicker healing.

**Materials and methods:**

This review compares the effectiveness of diode laser-assisted frenectomy in relation to tissue healing, patient discomfort, and clinical outcomes in comparison to scalpel frenectomy. Pertinent randomized controlled trials (RCTs) and observational studies were examined.

**Results:**

The results indicate that diode laser frenectomy is associated with less intraoperative bleeding and pain, and with similar long-term healing rates.

## Zusammenfassung

**Hintergrund:**

Die Frenektomie ist ein kieferchirurgischer Routineeingriff, bei dem ein abnormales Bändchen entfernt wird, um die Mundfunktion und die Ästhetik zu verbessern. Der Diodenlaser erfreut sich zunehmender Beliebtheit gegenüber der traditionellen Skalpellmethode, da er weniger postoperative Schmerzen, weniger Blutungen und eine schnellere Heilung verspricht.

**Methode:**

In der Übersicht wird die Wirksamkeit der Diodenlaser-gestützten Frenektomie in Bezug auf Gewebeheilung, Patientenbeschwerden und klinische Ergebnisse mit der Skalpell-Frenektomie verglichen. Es wurden randomisierte kontrollierte Studien (RCTs) und Beobachtungsstudien ausgewertet.

**Ergebnis:**

Die Ergebnisse weisen darauf hin, dass die Diodenlaser-Frenektomie mit weniger intraoperativen Blutungen und Schmerzen und mit ähnlichen langfristigen Heilungsraten verbunden ist.

## Introduction

Frenectomy is the surgical excision of the frenum. The frenum is an area of folded connective tissue that may have unfavorable effects, e.g., midline diastema, gingival recession, or speech difficulties. Conventional scalpel frenectomy, although effective, tends to cause postoperative bleeding and pain; it requires suturing. With recent developments in laser technology, diode lasers have emerged as a minimally invasive treatment, providing enhanced precision, less trauma, and faster healing [1].

In comparison with other lasers, for instance, like CO_2_, Nd:YAG, and Er:YAG, diode lasers are cost-effective, lightweight, and easy to handle, which makes them the first line of treatment for soft tissue management, such as frenectomy [2].

This review compares the clinical outcomes of diode laser-assisted frenectomy with conventional scalpel methods in terms of pain perception, wound healing, intraoperative bleeding, and patient satisfaction. The review also points out potential limitations and areas for future research.

## Materials and methods

A systematic PubMed, Scopus, Web of Science, and Cochrane Library database search was performed according to PRISMA guidelines. Articles published between 2015 and 2025 were eligible for inclusion. Included studies were randomized controlled trials (RCTs) and observational studies comparing diode laser frenectomy with traditional scalpel frenectomy.

Inclusion criteria were:

- Studies published from 2015 to 2025
- RCTs and observational studies
- Patients who underwent frenectomy using diode laser or scalpel
- Studies on postoperative pain, healing, bleeding, and patient satisfaction.

Exclusion criteria were:

- Patients with systemic illnesses interfering with wound healing
- Studies with missing data or uncertain method
- Animal studies and case reports.

Data extraction was done for postoperative pain, wound healing, bleeding, and patient satisfaction.

## Results

The final analysis included six studies comparing diode laser and scalpel frenectomy. The primary outcomes assessed were postoperative pain, wound healing, bleeding, and patient comfort. The summarized data from these studies are presented in Table 1 (Tab. 1).

## Discussion

The studies reviewed underscore that diode laser frenectomy causes much less postoperative pain than does scalpel frenectomy. Reduction in pain was noticed on the first postoperative day and continued for as long as one week after surgery [3], [4]. This is based on the fact that tissue ablation using laser seals nerve endings, which reduces inflammation and excitation of nociceptors. The laser-created heat also cauterizes sensory nerve endings, adding to diminished postoperative awareness of pain [5].

Wound healing was accelerated in the diode laser group in the initial week of healing, as shown by Singh et al. [6] and Fatima et al. [7]. At the 30-day follow-up, there was no difference in healing status between the two groups, which means that diode lasers are promoters of early healing but do not necessarily differ from the scalpel method in terms of long-term healing [8]. Comparative analysis revealed that the diode laser group experienced improved wound healing and lack of scar formation at 14 days after surgery because of the clean incision and minimal degree of wound contraction linked with the use of laser [9]. 

Laser-induced wounds heal with secondary intention and have minimal scarring in comparison to scalpel incisions. This is because of the reduced level of wound contraction after laser irradiation, which is achieved through the induction and development of fewer myofibroblasts and collagen fibers [10]. Uraz et al. [9], Derikvand et al. [11] and Azma et al. [12] also found similar results, indicating that diode laser frenectomy causes less swelling, bleeding, pain, and formation of scar tissue.

Intraoperative bleeding was found to be minimal in the diode laser group throughout. The coagulative effect of the laser eliminates the need for sutures and minimizes intraoperative blood loss, a significant benefit in operating rooms [13], [14]. Furthermore, diode laser-facilitated frenectomy takes less time during the procedure and reduces postoperative complications [3]. The controlled bleeding that can be obtained using diode lasers provides improved surgical accuracy and visibility [6].

Patient satisfaction and acceptance were significantly greater in the diode laser group in several studies. Lack of suturing, less postoperative pain, and quicker healing were reasons for this preference [8], [4]. Better esthetic outcomes and less scar formation also enhanced patient experience [7]. The diode laser method was linked with lower edema, better wound healing, and greater comfort/convenience in maintaining oral hygiene in comparison with the scalpel method [15].

The photothermal effect of diode lasers on tissues is responsible for their effectiveness in soft tissue surgery. The heat generated during the use of the laser occludes tiny blood vessels and lymphatic vessels, minimizing edema and intraoperative blood loss [5]. The absence of sutures in the diode laser group also enable improved oral hygiene and decreased plaque accumulation [15].

Closure of diastema following frenectomy was also examined in some studies. Tanik et al. [16] reported that after one-year of follow-up, diastema was reduced in both laser and scalpel groups; this indicates that the method chosen does not affect long-term closure of interdental space. Likewise, Ozener et al. [15] found no recurrence of frenulum attachment in either group, thus again proving the success of both the techniques. 

In spite of such advantages, some limitations were noted. Initial healing was quicker, but long-term results were similar in both techniques [8], [6]. The high expense of laser technology and the requirement for special training could also restrict its general use [13]. Some of the studies also mentioned that although diode lasers reduce discomfort, the quality of the outcome is operator-dependent [7]. The other limitation mentioned was the emission of fumes during the incision using the laser, giving off a smoky smell that can cause discomfort to the patient, making the use of a high-intensity air evacuator necessary [5]. In addition, the failure of diode lasers to fully incise muscle fibers from the periosteum promoted the likelihood of frenum reattachment in a few instances [4].

Lebret et al. [14] conducted an extensive systematic review including 10 studies and a total of 375 patients, which confirmed the existing evidence that diode lasers are superior to scalpel techniques in perioperative outcomes. Their findings confirmed significantly less postoperative pain, shorter surgery time, and fewer functional discomforts with laser use.

In comparison to the RCT by Eroglu et al. [3] and the observational study by Vincent et al. [4], the systematic review by Lebret et al. [14] demontrated that laser-assisted frenectomy offers better early healing and decreases intraoperative complications such as bleeding and suturing requirements [14]. Further studies indicate that while diode lasers accelerate short-term healing, there is no difference in long-term outcomes to those from scalpel techniques [8], [6]. The research mentioned above all points to the benefit of diode lasers in lessening immediate postoperative pain, but their superiority in the long term is not conclusive. 

## Conclusion

Diode laser frenectomy is a useful substitute for traditional scalpel frenectomy with benefits of less pain, less bleeding, and improved patient comfort. Although initial healing is quicker, long-term results are equivalent for both procedures. Cost and availability continue to be barriers to widespread use, as noted in smaller-scale research [13]. Long-term patient-reported outcomes and cost-benefit studies should be conducted in the future to determine the feasibility of diode laser frenectomy as a treatment of choice. Moreover, larger sample sizes and longer follow-up studies in the future are suggested to confirm these findings.

## Notes

### Authors’ ORCIDs

- Shunmugavelu K: 0000-0001-7562-8802
- Dhinakaran EC: 0000-0003-2194-6455

### Funding

None. 

### Competing interests

The authors declare that they have no competing interests.

## Figures and Tables

**Table 1:**
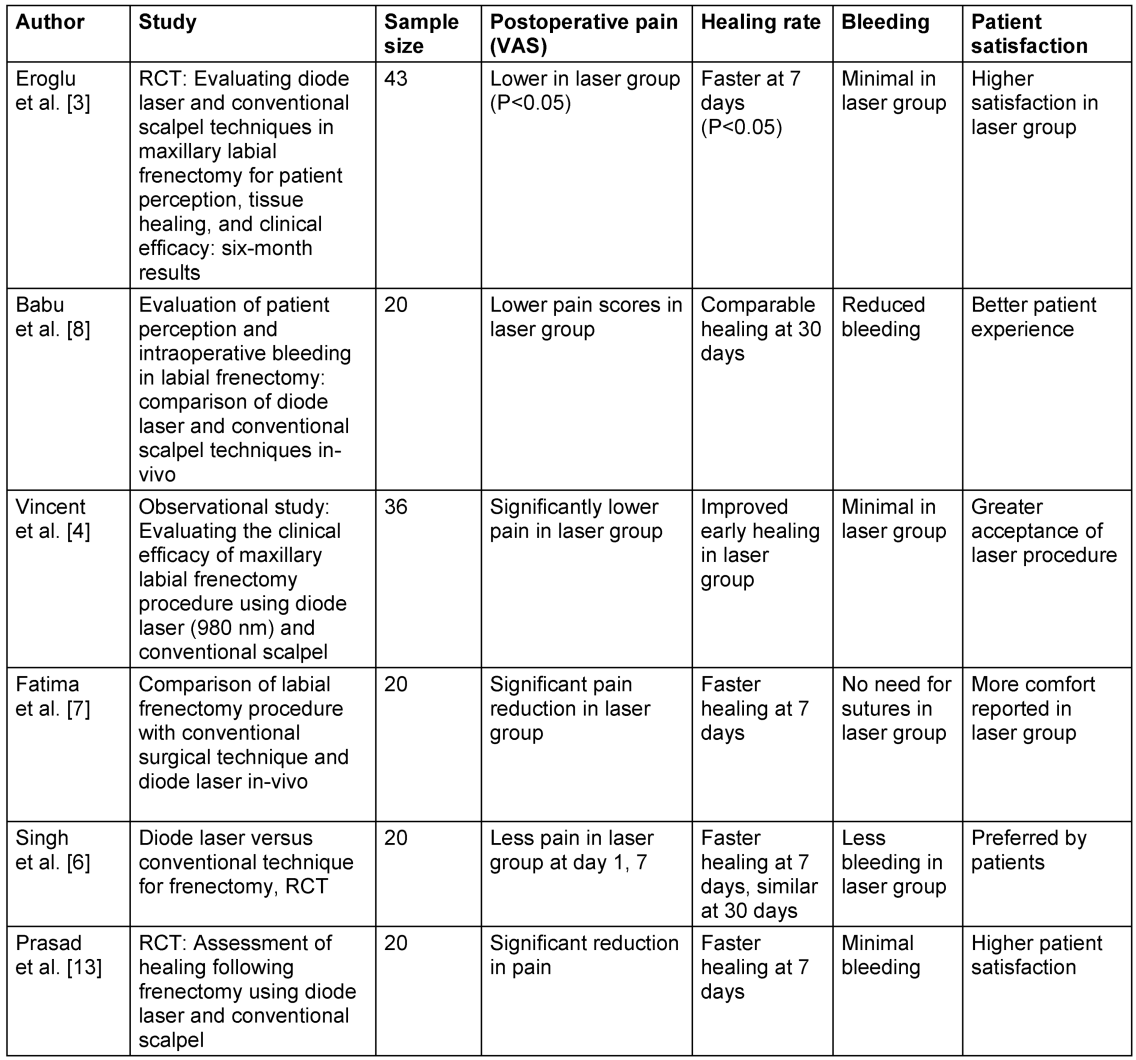
Summary of included studies
